# Neuronal Nicotinic Acetylcholine Receptors: Common Molecular Substrates of Nicotine and Alcohol Dependence

**DOI:** 10.3389/fpsyt.2013.00029

**Published:** 2013-04-30

**Authors:** Linzy M. Hendrickson, Melissa J. Guildford, Andrew R. Tapper

**Affiliations:** ^1^Department of Psychiatry, Brudnick Neuropsychiatric Research Institute, University of Massachusetts Medical SchoolWorcester, MA, USA

**Keywords:** nicotine, alcoholism, acetylcholine, nicotinic receptors, mesolimbic dopamine system

## Abstract

Alcohol and nicotine are often co-abused. As many as 80–95% of alcoholics are also smokers, suggesting that ethanol and nicotine, the primary addictive component of tobacco smoke, may functionally interact in the central nervous system and/or share a common mechanism of action. While nicotine initiates dependence by binding to and activating neuronal nicotinic acetylcholine receptors (nAChRs), ligand-gated cation channels normally activated by endogenous acetylcholine (ACh), ethanol is much less specific with the ability to modulate multiple gene products including those encoding voltage-gated ion channels, and excitatory/inhibitory neurotransmitter receptors. However, emerging data indicate that ethanol interacts with nAChRs, both directly and indirectly, in the mesocorticolimbic dopaminergic (DAergic) reward circuitry to affect brain reward systems. Like nicotine, ethanol activates DAergic neurons of the ventral tegmental area (VTA) which project to the nucleus accumbens (NAc). Blockade of VTA nAChRs reduces ethanol-mediated activation of DAergic neurons, NAc DA release, consumption, and operant responding for ethanol in rodents. Thus, ethanol may increase ACh release into the VTA driving activation of DAergic neurons through nAChRs. In addition, ethanol potentiates distinct nAChR subtype responses to ACh and nicotine *in vitro* and in DAergic neurons. The smoking cessation therapeutic and nAChR partial agonist, varenicline, reduces alcohol consumption in heavy drinking smokers and rodent models of alcohol consumption. Finally, single nucleotide polymorphisms in nAChR subunit genes are associated with alcohol dependence phenotypes and smoking behaviors in human populations. Together, results from pre-clinical, clinical, and genetic studies indicate that nAChRs may have an inherent role in the abusive properties of ethanol, as well as in nicotine and alcohol co-dependence.

## Introduction

Alcoholism is the third leading cause of preventable mortality in the world (Mokdad et al., 2004). Worldwide, about 2 billion people consume alcohol, with 76.3 million who have diagnosable alcohol use disorders (AUDs). Additionally, when analyzing the global burden of this disease, alcohol causes 2.5 million deaths per year (4% of the worldwide total) (World Health Organization, 2011). The estimated economic cost of alcoholism in the US alone, due to health care costs as well as productivity impacts such as lost wages, was $220 billion in 2005, which was significantly higher than cancer ($196 billion) or obesity ($133 billion) (CASA, 2000).

Interestingly, several reports from the 1980s to 1990s have estimated that 80% of alcohol-dependent people are also smokers (Bobo, 1992; Miller and Gold, 1998) and that smokers have an increased risk of developing AUDs (DiFranza and Guerrera, 1990; Grant et al., 2004). In addition, while the smoking rates in the general population of the U.S. have dramatically decreased over the past two decades, smoking has remained high in alcoholic individuals (Meyerhoff et al., 2006), with current estimates still between 70 and 75% (Bobo and Husten, 2000). These high rates of co-abuse of nicotine and alcohol have led some researchers to define this population as “alcoholic smokers” as compared to “smokers” (Littleton et al., 2007). Many hypotheses have been proposed as to the basis of the high rates of nicotine and alcohol co-abuse. For example, it is possible that alcohol use leads to nicotine use or vice versa (Tyndale, 2003), or that because alcohol and nicotine are legal and readily available, the likelihood of their co-use is increased (Funk et al., 2006). However, mounting genetic, pre-clinical, and clinical evidence indicates that neuronal nicotinic acetylcholine receptors (nAChRs), the molecular targets of nicotine that initiate dependence in smokers, may also contribute to alcohol’s abusive properties. In addition, neuronal nAChRs may represent common molecular targets where nicotine and ethanol functionally interact, potentially explaining the widespread co-morbidity between smoking and alcohol consumption. The focus of this review is to highlight this evidence, summarize recent findings, and identify gaps in knowledge regarding the role of nAChRs in alcohol dependence and nicotine and alcohol co-abuse.

## Neuronal nAChRs

Neuronal nAChRs are ligand-gated cation channels that are activated by the endogenous neurotransmitter acetylcholine (ACh) and the exogenous tertiary alkaloid nicotine (Albuquerque et al., 2009). They belong to the superfamily of Cys-loop ligand-gated ion channels that include receptors for γ-amino butyric acid (GABA, the GABA_A_, and GABA_C_ receptor), glycine, and 5-hydroxytryptamine (5-HT_3_) (Le Novere and Changeux, 1995; Changeux and Edelstein, 1998). These ligand-gated ion channels have similar structural and functional features. All subunits in this family contain a pair of disulfide-bonded cysteines separated by 13 residues (Cys-loop) in their extracellular amino terminus (Karlin, 2002).

**Figure 1:**
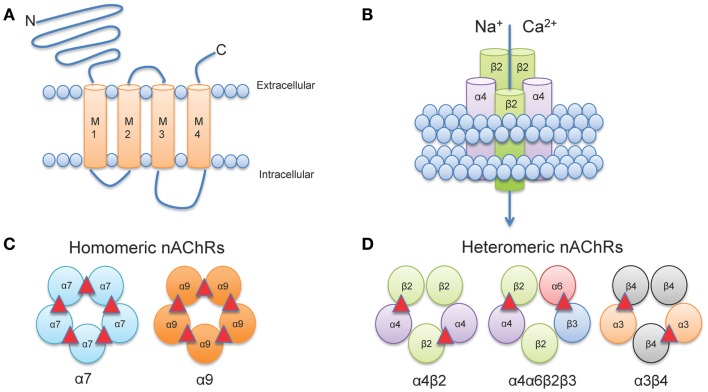
**Neuronal nAChR Structure**. **(A)** Membrane topology of a neuronal nAChR subunit. Each nAChR subunit contains four transmembrane domains (M1-M4), an extracellular amino- and carboxy-terminus, and a prominent M3-M4 intracellular loop of variable length. **(B)** Five subunits coassemble to form a functional subunit. **(C)** Homomeric receptors consist of α subunits only and usually have low affinity for agonist. To date, only mammalian α7, α9, and α10 (not shown) subunits may form functional homomers. **(D)** The majority of high affinity nAChRs are heteromeric and consist of a combination of α and β subunits. Importantly, multiple α subunits may coassemble with multiple β subunits in the pentameric nAChR complex (illustrated here by α4α6β3β2). ACh binding sites are depicted as red triangles.

Neuronal nAChRs, like all members of the cys-loop family of ligand-gated ion channels are formed by the arrangement of five subunits to create a central pore (Albuquerque et al., 2009). The structure of neuronal nAChRs is homologous to muscle nAChRs (Karlin, 2002), for which the atomic structure has been determined from electron microscopy studies from the fish electric organ (*Torpedo* nAChRs) (Miyazawa et al., 2003; Unwin, 2005). Each nAChR gene encodes a protein subunit consisting of a large amino-terminal extracellular domain composed of β-strands, four transmembrane α-helices segments (M1-M4), a variable intracellular loop between M3 and M4, and an extracellular carboxy-terminus (Corringer et al., 2000) (Figure 1A). The extracellular N-terminus contains the ACh binding domain that forms a hydrophobic pocket located between adjacent subunits in an assembled receptor (Sine, 2002). The M2 segment of all five subunits forms the conducting pore of the channel, and regions in the M2 intracellular loop contribute to cation selectivity and channel conductivity (Corringer et al., 2000) (Figure 1B).

In vertebrates, 12 genes encoding 12 distinct neuronal nAChR subunits have been identified (Cholinergic Receptor Nicotinic Alpha: CHRNA2-10 and Cholinergic Receptor Nicotinic Beta: CHRNB2-4 encoding α2-α10 and β2-β4 nAChR subunits, respectively all of which can be found in humans and other mammals, except for α8 which has only been identified in avian species (Millar and Gotti, 2009). Subunits are classified as either α-, by the presence of a Cys-Cys pair near the start of TM1, or non-α (β) when the Cys pair is missing (Le Novere and Changeux, 1995; Changeux and Edelstein, 1998).

Five subunits combine to form two classes of receptors: homomeric receptors containing only α subunits (α7-α9) or heteromeric receptors that contain α and β subunits (α2- α6 and β2-β4) (Dani and Bertrand, 2007) (Figures 1C,D). The most abundant subtypes in the brain are the low affinity α7 homomeric and high affinity α4β2* heteromeric nAChRs. An asterisk in nAChR nomenclature (i.e., α4*, α4β2*) indicates that other unidentified nAChR subunits may also be present and can be read as “α4 subunit containing nAChRs.” Importantly, heteromeric nAChRs are incredibly complex as they can contain two or three alpha subunits co-assembled with two or three beta subunits. For example, α4β2 nAChRs can be formed by either two α and three β subunits [(α4)_2_(β2)_3_] or three α and two β subunits [(α4)_3_(β2)_2_] (Zwart and Vijverberg, 1998; Nelson et al., 2003; Moroni et al., 2006). Each stoichiometry of the nAChR exhibits distinct sensitivity to agonist: [(α4)_2_(β2)_3_] nAChRs have a higher sensitivity to agonist (EC_50_ = ∼1 μM ACh); whereas [(α4)_3_(β2)_2_] nAChRs have a lower sensitivity to agonist (EC_50_ = ∼100 μM ACh) (Buisson and Bertrand, 2001; Nelson et al., 2003; Moroni et al., 2006). In addition, more than one type of alpha and/or beta subunit may be present in a functional receptor. For example, a subtype identified in midbrain dopaminergic (DAergic) neurons contains α4 and β2 subunits co-assembled with α6 and β3 subunits to form the α4α6β2β3* nAChR (Salminen et al., 2004, 2007; Zhao-Shea et al., 2011; Liu et al., 2012). This subunit diversity allows for a vast array of nAChR subtypes each with distinct pharmacological and biophysical properties (McGehee and Role, 1995; Gotti et al., 2007).

Neuronal nAChRs can exist in three conformational states and are regulated by exposure to agonist: closed at rest, when the receptor has low affinity for agonist and the channel is closed; the active state, when agonist occupies the ligand binding site and the channel is open allowing cations to flow down their electrochemical gradient; and the desensitized state, when the channel is occluded and the receptor is unresponsive to ligand (Dani and Bertrand, 2007; Albuquerque et al., 2009).

Interestingly, while nAChRs mediate fast, direct synaptic transmission at neuromuscular junctions and autonomic ganglia, there are very few examples of fast nicotinic transmission in the mammalian brain (Dani and Bertrand, 2007). However, neuronal nAChRs are expressed at the soma in neurons where they presumably modulate excitability directly. In addition, a significant proportion of nAChRs are located on presynaptic terminals (Role and Berg, 1996) where they facilitate Ca^2+^ dependent release of neurotransmitters (McGehee et al., 1995; Wonnacott, 1997). This may occur indirectly as a result of Na^+^ influx causing membrane depolarization and activation of voltage-gated Ca^2+^ channels or directly through Ca^2+^ influx through the channel itself (Albuquerque et al., 2009).

## Ethanol Modulation of Neuronal nAChRs: *In vitro* Studies

While ethanol modulates several ligand-gated ion channels including GABA_A_, NMDA, and 5-HT_3_ receptors (For a review see Spanagel, 2009), ethanol also potently modulates nAChRs at low concentrations of ethanol (100 μM–10 mM), identifying nAChRs as potential targets for ethanol action (Nagata et al., 1996). In heterologous expression systems, the effect of ethanol on nAChRs depends on the subunit composition of the nAChR. Expression of different combinations of human neuronal nAChR alpha and beta subunits in *Xenopus* oocytes, indicate acute ethanol (75 mM) potentiates ACh-induced current of α2β4, α4β4, α2β2, and α4β2 nAChRs while lower concentrations of ethanol (20–50 mM) inhibits nicotine-induced current of α7 nAChRs and all concentrations of ethanol tested have no effect on α3β2 or α3β4 nAChRs (Cardoso et al., 1999). Similar ethanol effects on heterologous expression of rat nAChRs in *Xenopus* oocytes have been observed except that ethanol could potentiate or inhibit α3β4 nAChRs at all ethanol concentrations tested likely reflecting oocyte batch to batch variability. In cultured rat cortical neurons, ACh-evoked nAChR currents insensitive to α-bungarotoxin (α-Bgtx), which blocks α7 nAChRs (i.e., heteromeric nAChRs) are significantly enhanced by physiologically relevant concentrations of ethanol while nAChRs sensitive to α-Bgtx (i.e., α7 homomeric nAChRs) are inhibited (Aistrup et al., 1999). Although not tested directly the α-Bgtx insensitive current profile was most similar to native α4β2* nAChRs (Marszalec et al., 1999).

Similar to other ligand-gated ion channels, ethanol potentiation of nAChRs is hypothesized to be a result of the ethanol-induced stabilization of the open channel state of the receptor (Wu et al., 1994; Forman and Zhou, 1999; Zuo et al., 2004). Site directed cysteine mutagenesis and covalent labeling with sulfhydryl reagents indicate that amino acid residues in the pore forming M2 region of neuronal nAChR at least partly contribute to the ethanol binding pocket (Borghese et al., 2002, 2003a,b). While individual amino acid residues forming the ethanol binding pocket may be distinct from other cys-loop receptors, the overall motif, the extracellular domain of M2, is critical for ethanol actions on nAChRs as well as GABA_A_ and glycine receptors (Borghese et al., 2003a). Additionally, it is possible that the ethanol-induced inhibitory effect seen with α7 nAChRs is due to the inherently fast desensitization rate of these receptors, implying that ethanol inhibition results in enhanced desensitization (Dopico and Lovinger, 2009). Thus, these and *in vivo* studies discussed below, suggest that ethanol modulation of nAChRs, either by enhancing or inhibiting function, may contribute to (1) the inherent mechanism of action of ethanol reward and (2) the common co-abuse of nicotine and alcohol.

## Neuronal nAChR Expression in the Mesocorticolimbic DA Pathway

Although neuronal nAChRs are expressed throughout the CNS, most studies focusing on the role of nAChRs in addiction have examined the mesocorticolimbic “reward” circuitry. Indeed, it is widely accepted that the mesocorticolimbic dopamine system plays a central role in modulating the rewarding effects of drugs of abuse (Wise and Bozarth, 1987; Koob, 1992).

**Figure 2:**
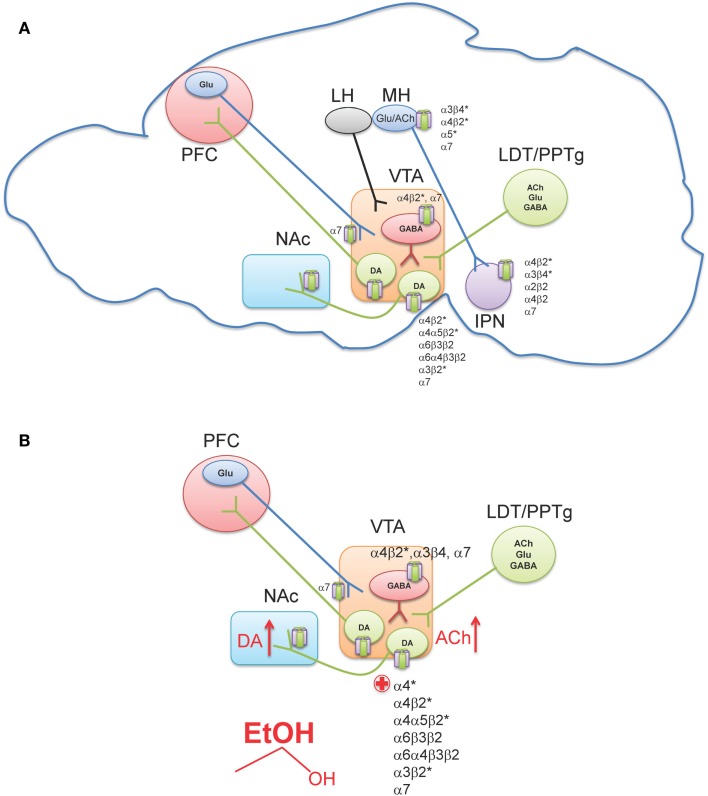
**Neuronal nAChR expression in the reward pathway**. **(A)** Sagittal rodent section illustrating the simplified mesocorticolimbic and habenulo-peduncular circuitry. Known neuronal nAChR subtypes expressed in different nuclei are indicated [for a review see (Millar and Gotti, 2009)]. **(B)** In the VTA, alcohol stimulates DAergic neurons at least, in part, via nAChR activation. Ethanol increases ACh release (red arrow, presumably through cholinergic projection from the LDT/PPTg) which in turn activates nAChRs on DAergic neurons driving activity. In addition, ethanol potentiates ACh activation at high affinity α4β2* nAChRs (red plus sign). The effect of alcohol on additional nAChRs in the VTA is unknown. This confluence of events in combination with other effects of alcohol in the VTA ultimately increases DA release in NAc (red arrow). VTA, Ventral tegmental area; NAc, Nucleus accumbens; PFC, Prefrontal cortex; LH, Lateral habenula; MH, Medial habenula; IPN, Interpeduncular nucleus; LDT, Lateral dorsal tegmentum; PPTg, Pedunculopontine tegmentum.

The ventral tegmental area (VTA) is located in the ventral midbrain, medial to the substantia nigra, and ventral to the red nucleus. It is referred to as an “area” and not considered to be a “nucleus” because the cryoarchitecture of the region is not well defined such that the boundaries of the VTA are determined by its neighboring structures (Fields et al., 2007; Ikemoto, 2007). Within the VTA are two main cell populations, DAergic projection neurons, which comprise ∼60% of cells in this region (Swanson, 1982), as well as local GABAergic interneurons and projection neurons (Carr and Sesack, 2000; Margolis et al., 2006a). The VTA receives inputs from regions throughout the CNS (Geisler and Zahm, 2005) including glutamatergic projections from the prefrontal cortex (PFC) (Sesack and Pickel, 1992), as well as glutamatergic, cholinergic, and GABAergic projections from two groups of mesopontine tegmental area neurons, the pedunculopontine tegmental nucleus (PPTg) and the laterodorsal tegmental nucleus (LDT; Figure 2A) (Cornwall et al., 1990; Semba and Fibiger, 1992; Oakman et al., 1995). Other regions projecting to the VTA include the nucleus accumbens (NAc), amygdala, ventral pallidum, superior colliculus, and lateral hypothalamus (For a review see Fields et al., 2007). Additionally, the lateral habenula (LH), a small nucleus that is a part of the epithalamus, has been shown to project to midbrain areas, and modulate the release of DA from the VTA and substantia nigra pars compacta (Herkenham and Nauta, 1979; Ji and Shepard, 2007; Matsumoto and Hikosaka, 2007).

Neurons in the VTA primarily project to the ventromedial striatum including the NAc shell and core as well as smaller projections to the PFC, hippocampus, entorhinal cortex, and lateral septal areas (Fields et al., 2007). Furthermore, studies using retrograde markers have shown that distinct groups of neurons originating in the VTA project to specific forebrain regions (Fallon et al., 1984; Margolis et al., 2006b). Projections to the NAc contain the largest proportion of DA neurons, with 65–85% being DAergic, while the PFC projections are only 30–40% DAergic (Swanson, 1982; Fallon et al., 1984). The remaining component of VTA afferents to the NAc and PFC contain GABAergic neurons (Carr and Sesack, 2000). The VTA is not a homogeneous region and can be divided into three sub-regions, the anterior VTA, posterior VTA, and the tail VTA. Additionally, evidence indicates that each region may project to distinct regions of the striatum and may also respond differently to drugs of abuse including nicotine and ethanol (Rodd et al., 2004a, 2010; Ikemoto, 2007; Shabat-Simon et al., 2008; Zhao-Shea et al., 2011). Importantly, nAChRs are robustly expressed in the VTA. DAergic neurons contain several nAChR subtypes including α4β2*, α4α5β2*, α4α6β2*, α6β2*, α3β2*, and α7 (Picciotto et al., 1998; Champtiaux et al., 2002; Marubio et al., 2003; Grady et al., 2007; Gotti et al., 2010; Zhao-Shea et al., 2011; Liu et al., 2012); whereas GABAergic VTA neurons express α4β2, α7, and α3β4 nAChRs (Figure 2A) (Klink et al., 2001; Mansvelder et al., 2002; Pidoplichko et al., 2004; Nashmi et al., 2007; Tolu et al., 2012).

## Neuronal nAChRs and Ethanol: *In vivo* Studies

The rewarding or reinforcing properties of ethanol and nicotine, as with most drugs of abuse, are associated with an increase in DA release in the NAc (Di Chiara and Imperato, 1988; Lewis and June, 1990; Benwell and Balfour, 1992; Samson et al., 1992; Diana et al., 1993; Weiss et al., 1993; Lanca, 1994; Pontieri et al., 1996). Both drugs increase the baseline firing frequency of VTA DAergic neurons and also increase the firing pattern from phasic to bursting, facilitating NAc DA release (Mereu et al., 1984; Gessa et al., 1985; Foddai et al., 2004; Exley et al., 2011; Li et al., 2011). Although the precise role of NAc DA release in reward is still under debate (Schultz, 2004; Salamone and Correa, 2012), ethanol- and nicotine-induced release of DA is critical for the onset and maintenance of dependence. Pharmacological blockade of DA receptors, destruction of DA neurons or lesioning of the NAc reduces ethanol and nicotine self-administration (Kiianmaa, 1978; Koob and Weiss, 1990; Corrigall and Coen, 1991; Corrigall et al., 1992, 1994; Rassnick et al., 1993; Ikemoto et al., 1997). In addition, rats will self-administer ethanol or nicotine directly into the VTA (Gatto et al., 1994; Ikemoto et al., 2006), and more specifically, the posterior VTA (Rodd et al., 2004b).

It is becoming increasingly clear that nicotine dependence is initiated by activation of DAergic neurons via nAChRs containing α4 and β2 subunits with some contribution of α6* nAChRs (Picciotto et al., 1998; Tapper et al., 2004; Maskos et al., 2005; Pons et al., 2008; Exley et al., 2011; Tolu et al., 2012). In the context of this review, we will not focus further on the mechanistic bases of nicotine dependence; rather we direct readers to a recent review article (De Biasi and Dani, 2011). In contrast to nicotine, multiple mechanisms underlying ethanol-mediated activation of VTA DAergic neurons have been proposed including modulation of intrinsic ion channels within these neurons, as well as ethanol-mediated alterations in synaptic input, both excitatory and inhibitory (Okamoto et al., 2006; Job et al., 2007; Xiao and Ye, 2008; Xiao et al., 2009; Rodd et al., 2010; Theile et al., 2011; Guan et al., 2012). However, cholinergic signaling through nAChRs also contributes to NAc DA release and ethanol reinforcement (Blomqvist et al., 1992, 1993, 1996; Ericson et al., 1998; Nadal et al., 1998; Dyr et al., 1999; Le et al., 2000; Soderpalm et al., 2000; Farook et al., 2009a; Kuzmin et al., 2009). One of the most consistent findings implicating nAChRs in ethanol behaviors associated with reward/reinforcement is that the non-specific nAChR antagonist, mecamylamine, reduces ethanol consumption and blocks ethanol-induced DA release in the NAc. Originally discovered by pioneering work of Soderpalm and Engel, systemic mecamylamine significantly reduces ethanol-mediated extracellular DA release in the NAc (Blomqvist et al., 1993), and reduces ethanol consumption in rats (Blomqvist et al., 1996). The effect of mecamylamine is localized to the VTA, as local infusion of the antagonist in rat midbrain but not NAc reduces NAc DA release elicited by ethanol (Blomqvist et al., 1997). VTA infusion of mecamylamine also reduces rat operant responding for ethanol and ethanol-associated cues, as well as consumption during relapse (Lof et al., 2007; Kuzmin et al., 2009). In mice, mecamylamine delivered systemically reduces ethanol consumption in C57Bl/6J mice in the restricted access ethanol consumption “drinking in the dark” (DID) paradigm (Hendrickson et al., 2009), a model of binge drinking (Rhodes et al., 2005, 2007), as well as in the two-bottle choice consumption assay (Farook et al., 2009a). What is mecamylamine’s mechanism of action in reducing ethanol consumption? In mice, mecamylamine apparently blocks activation of VTA DAergic neurons by ethanol as measured by c-Fos induction after challenge with an intraperitoneal injection (i.p.) of ethanol (Hendrickson et al., 2009). More recently, it has been demonstrated that mecamylamine blocks ethanol-mediated activation of VTA DAergic neurons in mouse midbrain slices (Liu et al., 2013). Mecamylamine also blocks the ability of ethanol to condition a place preference in mice (Bhutada et al., 2012). Thus, these data suggest that nAChR expressed in the VTA contribute to ethanol activation of DAergic neurons and ethanol reward. The effects of mecamylamine in these pre-clinical models may have predictive validity as patients administered mecamylamine report reduced pleasurable effects of alcoholic beverages (Chi and de Wit, 2003). As discussed above, ethanol is not a direct agonist at nAChRs; rather it potentiates or inhibits nAChRs depending on subtype. Thus, nAChR involvement in ethanol reward implies that ethanol must increase ACh concentrations in brain regions involved in reward/reinforcement. To date, one study has measured extracellular concentrations of ACh in the VTA of rats that voluntarily consumed ethanol and found that ACh levels were increased after ethanol consumption and shortly thereafter, DA concentrations were elevated in the NAc as well (Larsson et al., 2005). These data indicate that the increase in VTA ACh could drive activation of DAergic neurons through nAChRs (Figure 2B). While the predominant VTA cholinergic afferents project from the PPTg and LDT area (Oakman et al., 1995), brain regions that have also been implicated in mediating natural as well as drug-reward behavior (Yeomans et al., 1993), additional experiments will be needed to verify that these inputs mediate ethanol-induced increases in VTA ACh. In addition, the mechanism by which ethanol could elicit an increase in ACh release into the VTA is unknown and warrants further study.

## Neuronal nAChRs and Alcohol: Identifying Relevant Subtypes: Pharmacology

Because mecamylamine blocks virtually all subtypes of nAChRs, it provides little insight into the subunit composition of key nAChRs involved in ethanol activation of DAergic neurons or ethanol behaviors associated with the VTA such as consumption. Thus, several studies have used additional, more selective nAChR antagonists, in an effort to uncover the nAChR subtype(s) that may be involved in ethanol’s mechanism of action (Table 1). Studies in VTA responses to *nicotine* indicate that DAergic neurons contain several nAChR subtypes including α4β2*, α4α5β2*, α4α6β2*, α6β2*, α3β2*, and α7 (Picciotto et al., 1998; Champtiaux et al., 2002; Marubio et al., 2003; Grady et al., 2007; Gotti et al., 2010; Zhao-Shea et al., 2011; Liu et al., 2012). Identifying the precise subunit composition of nAChRs involved in ethanol consumption and activation of VTA DAergic neurons is challenging due to the sheer number of potential subunit combinations that may be expressed in the VTA. However, identifying one or more nAChR subtypes involved in ethanol activation of VTA and/or reward may lead to novel targets to reduce consumption. Systemic injection or VTA infusion of the competitive α4β2 nAChR antagonist, dihydro-β-erythroidine (DHβE), in rats, fails to reduce ethanol-mediated DA release in the NAc and ethanol intake (Ericson et al., 2003; Chatterjee et al., 2011). In addition, low doses of DHβE also have little effect on operant responding for ethanol in rats, although a higher dose can reduce responding (Kuzmin et al., 2009). Systemic injection of DHβE does not reduce consumption in mice as measured in the DID assay nor ethanol-induced NAc DA release (Larsson et al., 2002; Hendrickson et al., 2009). Together these data suggest that α4β2 nAChRs may not be critical for ethanol reward and consumption behavior. However, sensitivity of α4β2* nAChR blockade by DHβE is dependent on the stoichiometry of the receptor and the expression of other non-α4β2 subunits that may also be present in an α4β2* nAChR complex (Harvey and Luetje, 1996; Harvey et al., 1996; Le et al., 2000; Larsson et al., 2002; Ericson et al., 2003; Moroni et al., 2006; Lof et al., 2007; Kamens and Phillips, 2008). The α7 selective antagonist, methyllycaconitine (MLA), does not affect ethanol-mediated behaviors including consumption, ethanol-induced DA release in NAc and ethanol operant responding in rats, as well as, consumption in mice. While caution with interpretation of these results is warranted due to data indicating higher concentrations of MLA may also antagonize non-α7 nAChRs (of an unknown nAChR subtype that may include α6 and/or α3 subunits (Mogg et al., 2002)), homomeric α7 nAChRs may not be involved in ethanol reinforcement (Larsson et al., 2002; Hendrickson et al., 2009; Kuzmin et al., 2009). On the other hand, the α3β2*, β3*, and α6* subtype-selective antagonist, α-conotoxin MII (Cartier et al., 1996), when infused into the VTA does inhibit ethanol consumption, operant responding, and DA release in the NAc of rats (Larsson et al., 2004, 2005; Kuzmin et al., 2009) and reduce ethanol-induced locomotor stimulation and increases in NAc DA release in mice (Larsson et al., 2004; Jerlhag et al., 2006). Importantly, recent data indicate that approximately half of α-conotoxin MII-sensitive nAChRs in the striatum also contain the α4 subunit (Grady et al., 2007; Salminen et al., 2007) and deletion of β2* nAChRs nearly abolishes α-conotoxin MII binding in the VTA (Marubio et al., 2003). However, infusion of α-conotoxin PIA, which may have more selectivity for α6* nAChRs than α3* nAChRs (Dowell et al., 2003), failed to reduce ethanol-induced DA release in NAc when infused in the VTA suggesting that α3* nAChRs may be more critical for ethanol reward. Finally, systemic injection of the α3β4* nAChR-selective antagonist 18-methoxycoranaridine (18-MC) reduces ethanol consumption in alcohol-preferring rats (Rezvani et al., 1997). However, direct infusion of 18-MC into the VTA fails to reduce alcohol consumption (Carnicella et al., 2010) in rats consistent with data indicating low expression of β4* nAChRs in VTA DAergic neurons (Gotti et al., 2010; Zhao-Shea et al., 2011).

**Table 1:** **Neuronal nAChR ligands that modulate alcohol behaviors**.

Drug	nAChR subtype target	Route of delivery	Effect on ethanol behavior (in rodents)
Mecamylamine	Non-selective antagonist	i.p.	Decreased ethanol intake in rats (Blomqvist et al., 1996)
		i.p.	Decreased ethanol intake in mice (Hendrickson et al., 2009)
		i.p.	Blocked ethanol-induced DA release in NAc in rats (Blomqvist et al., 1993)
		i.p.	Partially counteracted ethanol-induced enhancements of locomotor activity and brain DA turnover in mice (Blomqvist et al., 1992)
		i.p.	Blocked ethanol-induced activation of DA neurons in mice (Hendrickson et al., 2009)
		i.p.	Reduced operant self-administration and blocked deprivation-induced increase in alcohol consumption in rats (Kuzmin et al., 2009)
		VTA	Reduced ethanol-induced accumbal DA release in rats (Ericson et al., 1998)
		i.p.	Reduced ethanol intake in rats (Le et al., 2000)
Nicotine	Agonist	s.c. (chronic)	Increased ethanol intake in rats (Potthoff et al., 1983; Le et al., 2000)
		s.c. (subchronic/acute)	Increased ethanol intake in rats (Blomqvist et al., 1996; Le et al., 2000)
		s.c. (subchronic)	Increased ethanol preference in rats (Blomqvist et al., 1996)
		s.c. (acute)	Enhanced ethanol-induced locomotor stimulation in mice (Blomqvist et al., 1992)
		s.c. (subchronic)	Enhanced ethanol-induced locomotor stimulation in rats (Blomqvist et al., 1996)
		s.c. (subchronic)	Enhanced DA turnover-increasing effect of ethanol in rats (Johnson et al., 1995)
		s.c. (chronic)	Decreased ethanol intake in rats (Sharpe and Samson, 2002)
		s.c. (chronic)	Decreased ethanol seeking in rats (Sharpe and Samson, 2002)
		i.p. (acute)	Decreased ethanol intake in mice (Hendrickson et al., 2011)
Varenicline	α4β2 Partial agonist high affinity α3β2, α3β4, α6*, α7 low affinity binding	i.p. and VTA	Decreased ethanol intake in mice (Hendrickson et al., 2010; Kamens et al., 2010; Santos et al., 2012)
		i.p.	Decreased ethanol intake in rats (Steensland et al., 2007)
		i.p.	Reduced ethanol seeking and consumption with no rebound increase in ethanol after cessation in rats (Steensland et al., 2007)
		i.p.	Reduced operant ethanol self-administration and blocked deprivation-induced relapse-like consumption in rats (Kuzmin et al., 2009)
		s.c.	Blocks increase in extracellular DA in NAc following acute ethanol injection in rats (Ericson et al., 2009)
α-Conotoxin MII	α6*, α3β2* Antagonist	VTA	Reduced alcohol-induced DA release in mice (Larsson et al., 2004)
		VTA	Reduced locomotor stimulation in mice (Larsson et al., 2004)
		VTA	Decreased self-administration of ethanol in rats (Kuzmin et al., 2009)
		VTA	Blocked deprivation-induced relapse-like ethanol consumption in rats (Kuzmin et al., 2009)
DHβE	α4β2* antagonist	s.c.	No effect on ethanol consumption in rats (Le et al., 2000)
		s.c.	No effect on DA-enhancing effect of ethanol in mice (Larsson et al., 2002)
		i.p.	Inhibited ethanol intake at 4mg/kg in rats (Kuzmin et al., 2009)
		s.c.	No effect on ethanol consumption in rats (Chatterjee et al., 2011)
MLA	α7* antagonist	i.p.	No effect on DA-enhancing effect of ethanol in mice (Larsson et al., 2002)
		i.p.	No effect on self-administration of ethanol or deprivation-induced relapse-like drinking in rats (Kuzmin et al., 2009)
		i.p.	No effect on ethanol consumption in DID in mice (Hendrickson et al., 2009)
α-Conotoxin PIA	α6* antagonist	VTA	No effect on ethanol-induced locomotor stimulation or enhanced DA release in mice (Jerlhag et al., 2006)
CP-601932	α3β4 and α4β2 high affinity partial agonist	s.c.	Decreased ethanol consumption and operant self-administration in rats (Chatterjee et al., 2011)
PF-4575180	α3β4 high affinity partial agonist	s.c.	Decreased ethanol consumption and operant self-administration in rats (Chatterjee et al., 2011)
Lobeline	Non-selective antagonist, particularly at β2* nAChRs	s.c.	Reduced ethanol consumption in DID and during continuous ethanol access in mice (Farook et al., 2009b; Sajja and Rahman, 2011)
		s.c.	Reduced ethanol-induced DA and its metabolite levels in ventral striatum in mice (Sajja et al., 2010)
Cytisine	Low-efficacy partial agonist with high affinity for α4β2* nAChRs. Full agonist at β4* and α7* nAChRs	s.c.	Reduced ethanol consumption in DID in mice and during continuous ethanol access in mice (Hendrickson et al., 2009; Sajja and Rahman, 2011)
		s.c.	Reduced ethanol-induced DA and its metabolite in mice (Sajja et al., 2010)
Sazetidine-A	Highly selective α4β2 desensitizer	s.c.	Reduces alcohol intake in rats (Rezvani et al., 2010)

## Neuronal nAChRs and Alcohol: Identifying Relevant Subtypes: Mouse Genetics

Behavioral studies in genetically engineered mice have also been used to glean information on nAChR subtypes that may be involved in alcohol consumption and reward. Mice that do not express chrnb2, the gene encoding the nAChR β2 subunit (β2 KO) consume and prefer ethanol in a 24 h access two-bottle choice paradigm similar to wild-type (WT) littermates indicating that β2* nAChR may not play a role in baseline ethanol consumption in this assay (Kamens et al., 2010). Similarly, α6 KO and β3 KO mice consume ethanol similar to WT in a 24 h access two-bottle choice consumption assay (Kamens et al., 2012). Female α7 KO mice consume significantly less ethanol in this paradigm compared to female WT littermates; whereas male α7 KO and WT mice consume similar amounts of ethanol indicating a potential gender effect of α7 nAChRs on ethanol consumption (Kamens et al., 2010). α5 KO mice do not differ in acute ethanol consumption, as measured by the DID assay, compared to WT (Santos et al., 2012). Together, these data indicate that nAChRs containing α5, α6, β2, or β3 subunits may not be critical for ethanol consumption *per se*. However, as nAChRs are robustly expressed in a variety of brain regions, subunit compensation may occur in a KO mouse background (Drago et al., 2003). Thus, these results will need to be verified using shRNAs to knock-down nAChR subunits in discreet brain regions. Interestingly, sleep time elicited by high doses of ethanol is increased in α6 and α5, but not β3 KO mice compared to their WT littermates indicating a role for α6* and α5* nAChR in alcohol-induced sedation (Kamens et al., 2012; Santos et al., 2012).

In contrast to the majority of KO models discussed above, acute ethanol consumption in the DID paradigm is significantly less in α4 KO mice compared to WT for high (20%) but not low (2%) concentrations of ethanol implicating a role for α4* nAChR in ethanol consumption (Hendrickson et al., 2010, 2011). In addition, the ability of ethanol to condition a place preference in α4 KO mice is reduced compared to WT. Conversely, in mice harboring a point mutation in α4* nAChRs that renders receptors hypersensitive to agonist [the Leu9′Ala α4 knock-in line (Tapper et al., 2004; Fonck et al., 2005)], a sub-threshold dose of ethanol is sufficient to condition a place preference indicating that α4* nAChRs modulate alcohol reward (Liu et al., 2013). Consistent with behavioral data, ethanol activation of VTA DAergic neurons is reduced in α4 KO midbrain slices and more robust in Leu9′Ala midbrain slices. Finally, ethanol potentiates the response to bath applied ACh in midbrain DAergic neurons and potentiation is abolished in DAergic neurons of α4 KO mice (Liu et al., 2013). Together, these data indicate that α4* nAChRs in VTA DAergic neurons may contribute to ethanol activation of the VTA and alcohol reward although additional experiments are needed to confirm that the observed difference in ethanol-mediated behaviors in these mouse models are due to α4* nAChRs in the VTA as these receptors are expressed throughout the CNS (Baddick and Marks, 2011).

## Nicotine and Alcohol Interactions: *In vivo* Studies

Human studies have shown that individuals dependent on alcohol have higher rates of nicotine dependence (Room, 2004), and smokers tend to consume more ethanol than non-smoking alcohol users (York and Hirsch, 1995). Unlike the majority of clinical studies, nicotine administration can either increase ethanol intake (Potthoff et al., 1983; Blomqvist et al., 1996; Smith et al., 1999; Le et al., 2000; Clark et al., 2001; Ericson et al., 2003), or decrease ethanol intake (Nadal et al., 1998; Dyr et al., 1999; Sharpe and Samson, 2002) in rats. These conflicting results have led to a complex and interesting questions: under what conditions (i.e., time delay between nicotine and ethanol, dose of nicotine, length of ethanol presentation, acute versus chronic nicotine/ethanol etc.) does nicotine increase ethanol intake, and what conditions cause a decrease in ethanol intake?

Blomqvist et al. (1996) demonstrated that daily nicotine during ethanol deprivation and ethanol reinstatement increases ethanol intake and preference in rats shown to have a medium baseline preference (25–65%) for ethanol over water. Similarly, Le et al. (2003) demonstrated that rats increased lever presses for ethanol during the course of daily nicotine injection paired 15 min prior to an operant session. These data are in agreement with various other experiments in which nicotine was given either constantly or repeatedly (Potthoff et al., 1983; Smith et al., 1999; Ericson et al., 2000; Olausson et al., 2001). In rats, nicotine can also reinstate alcohol seeking after extinction and increase ethanol self-administration when administered during an ethanol deprivation period (Lopez-Moreno et al., 2004). Interestingly, rats given nicotine only during the relapse period, once self-administration has resumed after a deprivation period, consume less ethanol, and rats given nicotine during both abstinence and relapse increased ethanol intake compared to control (Alen et al., 2009).

In contrast, Sharpe and Samson demonstrated that ethanol intake and lever pressing during operant ethanol self-administration are both decreased after a high dose of nicotine (0.7 mg/kg, subcutaneous injection (s.c.), expressed as free base nicotine) 30 min prior to ethanol self-administration, and with a lower dose of nicotine (0.35 mg/kg, s.c.). While locomotor depression by nicotine could potentially confound the interpretation of decreased ethanol self-administration, this is unlikely as nicotine injections did not decrease sucrose self-administration. Thus, Sharpe and Samson (2002) propose that nicotine could be acting as a reinforcer of ethanol, decreasing the amount of ethanol necessary to achieve satiety. This is in agreement with other studies in which nicotine is administered either immediately prior to, or within 30 min of, ethanol presentation or self-administration (Nadal et al., 1998; Damaj, 2001).

To reconcile differences in nicotine effects on ethanol consumption and self-administration, Hauser et al. demonstrated that acute nicotine administration affects ethanol seeking and relapse in a time-dependent manner. Nicotine injection immediately prior to an ethanol operant self-administration session in ethanol preferring rats elicits reduced responding for ethanol compared to a saline injection; whereas nicotine exposure 4 h prior will increase responses (Hauser et al., 2012). These data indicate that acute nicotine may initially act as a substitute for ethanol at the immediate time-point causing a reduction in craving for ethanol and, at the later time-point, nicotine may lead to desensitization of nAChRs in the brain, enhancing ethanol seeking.

As in rats, acute nicotine immediately prior to presentation of ethanol in the DID paradigm reduces consumption in mice (Hendrickson et al., 2009); whereas chronic nicotine treatment increases consumption (Sajja and Rahman, 2012). The reduction of ethanol consumption is mediated by nAChRs containing the α4 subunit: nicotine fails to reduce consumption in α4 KO mice; whereas acute sub-threshold nicotine doses are sufficient to reduce consumption in Leu9′Ala mice (Hendrickson et al., 2011). The effect of acute nicotine activates the posterior VTA as measured by increased c-Fos in mouse VTA DAergic neurons while an additional injection of ethanol does not further activate these neurons, consistent with nicotine substituting for ethanol during this treatment schedule (Hendrickson et al., 2009).

The mechanistic basis of chronic nicotine on ethanol consumption is unclear. However, nicotine potentiates the response to ethanol in VTA DAergic neurons (Clark and Little, 2004) and repeated nicotine infusion into the posterior VTA increases the stimulatory effects of ethanol (Ding et al., 2012). These data indicate that chronic nicotine treatment may actually increase the reinforcing/rewarding properties of alcohol. Interestingly, chronic nicotine upregulates midbrain nAChRs which may lead to increased DAergic neuron activation by ethanol (Nashmi et al., 2007).

## Neuronal nAChR Ligands for Reducing Ethanol Consumption

While several areas of alcoholism research exist, the end goal of the majority of research is to identify new and improved treatment options for those suffering from alcoholism. Currently, there are three FDA approved medications for treating alcoholism. The first, disulfiram, was approved in 1954, and is classified as an anti-relapse medication (Christensen et al., 1991). It is an acetaldehyde dehydrogenase inhibitor, which after drinking alcohol allows the buildup of acetaldehyde in the blood, causing symptoms including headache, nausea, vomiting, weakness, mental confusion, or anxiety (Christensen et al., 1991). However, in recent years, many physicians have stopped prescribing this drug because of the severe symptoms it causes and the fact that if a patient wished to drink again, they could simply not take their medication. Naltrexone, available since 1994, is a competitive opioid receptor antagonist that works by decreasing the euphoric effects produced by alcohol. It is considered to be an anti-relapsing drug because it decreases heavy drinking in patients with alcoholism and prevents relapse to heaving drinking (O’Malley et al., 1992; Volpicelli et al., 1992). The third drug, acamprosate, is a partial agonist of NMDA glutamate receptors and an antagonist of metabotropic glutamate receptors and is thought to act as an anti-craving medication by inhibiting glutamate signaling (Mason, 2003; Mason et al., 2006). While European studies have reported modest benefits with acamprosate, these studies have not been reproducible in the US (Pettinati et al., 2006).

Unfortunately, while these medications have been effective for some, only 20–30% of treated patients respond to the anti-craving and anti-relapsing compounds (Spanagel, 2009). Interestingly, new studies have shown that people with different genetic profiles may drink for different reasons, and also that they may respond better to one type of medication versus another. For example, populations with a specific type of mu opioid receptor respond to naltrexone better than others, and this group has been described as “feel good drinkers” (Oslin et al., 2006; Anton et al., 2008). Another population of alcoholics report that they drink to relieve feelings of stress and anxiety (Kuehn, 2009) for which new medications are currently being tested (George et al., 2008). This large variability in patient response is a driving force in identifying new molecular targets for improved pharmacotherapeutic drugs. Consequently, the main focus of alcoholism treatments has been to restore the balance to the different biochemical pathways in the brain that are disrupted during alcohol dependence.

Varenicline, an α4β2 partial agonist clinically approved as a smoking cessation therapeutic (Coe et al., 2005; Gonzales et al., 2006; Jorenby et al., 2006; Tonstad et al., 2006; Steensland et al., 2007), can reduce ethanol intake, ethanol seeking, and cue-induced ethanol reinstatement in rats (Steensland et al., 2007; Wouda et al., 2011) and ethanol consumption in mice (Hendrickson et al., 2010; Kamens et al., 2010; Santos et al., 2012). In addition, varenicline can also reduce the enhancing effect of chronic nicotine on ethanol self-administration in rats (Bito-Onon et al., 2011). Coupled with clinical data indicating that varenicline reduces ethanol consumption in heavy drinking smokers (McKee et al., 2009; Fucito et al., 2011; Mitchell et al., 2012), uncovering the mechanism of action of varenicline could lead to more refined nAChR partial agonists for the treatment of alcoholism. In mice, systemic injection of lower doses of varenicline immediately prior to ethanol bottle presentation reduces ethanol consumption in the DID paradigm (Hendrickson et al., 2010). In addition, this effect of varenicline is reduced in α4 KO mice and enhanced in mice that express α4* nAChR that are hypersensitive to agonist indicating that activation of α4* nAChR may underlie varenicline effects on binge drinking. However, while varenicline was designed to be selective for α4β2* nAChRs at low doses, at high concentrations, varenicline is also a partial agonist at α6β2* nAChRs, a full agonist at α3β4 and α7 nAChRs, as well as at 5-HT_3_ receptors (Mihalak et al., 2006; Papke et al., 2010; Lummis et al., 2011; Bordia et al., 2012), which may also explain some of its effects on ethanol consumption especially in response to high doses used to reduce ethanol preference and seeking in most studies using the two-bottle choice 24 h access paradigm of ethanol consumption. Indeed, varenicline still reduces ethanol consumption in β2 and α7 KO mice (Kamens et al., 2010). Varenicline also reduces ethanol consumption in the DID paradigm in α5 KO mice (Santos et al., 2012). Thus, the mechanism of varenicline induced reduction in ethanol consumption and the nAChR subtype responsible for this effect is still unclear. However, acutely, varenicline reduces ethanol-mediated DA release in NAc of rats, an effect that diminishes with repeated exposure of the partial agonist (Ericson et al., 2009), consistent with varenicline reducing the rewarding properties of ethanol. In contrast, a recent clinical study found that varenicline potentiated aversion to ethanol in social drinkers (Childs et al., 2012), suggesting the agonist may reduce consumption through an anti-reward pathway.

In addition to varenicline, pre-clinical data are emerging regarding other nAChR ligands that may prove effective in reducing ethanol consumption. Sazetidine-A, an α4β2* nAChR-selective “desensitizer” and partial agonist can reduces ethanol consumption in rats (Rezvani et al., 2010). Lobeline, an antagonist with high affinity for α4β2* and α3β2* nAChRs reduces ethanol consumption in mice in the DID and two-bottle choice paradigm (Farook et al., 2009b). Cytisine, a partial agonist that preferentially activates high affinity β2* nAChRs at low doses but also is a full β4* nAChR agonist at high doses also reduces ethanol consumption (Bell et al., 2009; Hendrickson et al., 2009; Sajja and Rahman, 2011, 2012). Both lobeline and cytisine reduced ethanol-mediated DA release in ventral striatum of mice consistent with blocking of ethanol reward/reinforcement (Sajja et al., 2010). In addition, lobeline and cytisine also reduce the increase in alcohol consumption that occurs with chronic nicotine exposure in the DID paradigm (Sajja and Rahman, 2012). Finally, novel partial agonists targeting α3β4* nAChRs reduce ethanol consumption and seeking in rats (Chatterjee et al., 2011).

## Neuronal nAChR Subunit Genes and Alcohol: Human Genetic Association Studies

There is growing evidence that suggests that common genes may influence the development of alcohol and nicotine behaviors individually as well as contribute to both disorders in humans (True et al., 1999; Bierut et al., 2000; Madden and Heath, 2002). Using twin studies, it was determined that identical twins are two times as likely to be dependent on alcohol and/or nicotine if the other twin is dependent, compared to fraternal twins (Heath et al., 1997).

Recent genome wide association studies have identified several polymorphisms within genetic loci that includes the nAChR subunit genes CHRNA5/A3/B4 (which encode the nAChR α5, α3, β4 subunit, respectively), that are associated with nicotine dependence, COPD, and lung cancer (Amos et al., 2008; Berrettini et al., 2008; Bierut et al., 2008; Hung et al., 2008; Saccone et al., 2010). Interestingly, genetic variation in these genes has also been associated with age of initiation of smoking and alcohol use and level of response of alcohol use (Joslyn et al., 2008; Schlaepfer et al., 2008). Two SNPs associated with nicotine dependence and lung cancer have been found to also be associated with a low level of response to alcohol, a phenotype considered a risk factor for likelihood of developing an AUD (Joslyn et al., 2008). Thus, common SNPs may confer susceptibility to both nicotine dependence and alcoholism. In addition, genetic variation in CHRNA5, distinct from those associated with nicotine dependence, are also associated with alcohol dependence (Wang et al., 2009). The mechanistic bases for how polymorphisms in CHRNA5/A3/B4 modulate nicotine and alcohol phenotypes are unclear although distinct SNPs in CHRNA5 have been shown to affect α4β2 nAChR function *in vitro* and mRNA expression in human brain (Bierut et al., 2008; Wang et al., 2009). It is also unclear if genetic variation in CHRNA5/A3/B4 is specific for modulation of nicotine and alcohol dependence as SNPs are also associated with cocaine and opioid dependence, as well as substance use initiation (Grucza et al., 2008; Sherva et al., 2010; Lubke et al., 2012; but see Chen et al., 2012). Thus, SNPs in this region may affect aspects of addiction common to all drugs of abuse, such as reward, tolerance, or withdrawal. Alternatively, CHRNA5/A3/B4 may play a role in general risk taking behavior or impulsivity which may significantly predispose one to drug addiction (Stephens et al., 2012).

Additional genes encoding nAChR subunits have been linked to alcohol phenotype. SNPs in CHRNB2, have been associated with the subjective responses to both alcohol and nicotine (Ehringer et al., 2007); whereas only a modest association of alcohol responses with CHRNA4 SNPs were reported. An additional study identified a CHRNA4 SNP associated with alcoholism in a small Korean population (Kim et al., 2004). Finally, SNPs within CHRNA6 and CHRNB3 are associated with heavy alcohol consumption (Hoft et al., 2009; Landgren et al., 2009), as well as smoking behavior (Thorgeirsson et al., 2010).

Together these human genetic studies indicate that heritable polymorphisms within nAChR subunit genes may predispose distinct populations to increased risk for AUDs and, perhaps nicotine and alcohol co-dependence.

## Future Directions

Emerging evidence indicates that SNPs within genes encoding nAChR subunits are associated with alcohol dependence phenotypes. Additional research is needed to understand how SNPs in these subunits modulate the effects of ethanol on nAChRs directly and in animal models of ethanol dependence. It will also be critical to expand the focus of nAChRs and ethanol effects on circuits outside of the mesocorticolimbic pathway. Indeed, recent data indicate that nicotine intake is controlled by the habenulo-peduncular axis. This circuit consists of a small, epithalamic structure, the habenula (Hb) which can be divided into medial (MH) and lateral (LH) sub-regions (Hikosaka, 2010). The Hb projects to its target brain regions through a conspicuous bundle of axons that make up the fasciculus retroflexus. The LH projects to the rostromedial tegmental nucleus that is involved in the modulation of DA release from the susbstantia nigra pars compacta and VTA (Kaufling et al., 2009; Bromberg-Martin et al., 2010a,b; Balcita-Pedicino et al., 2011; Hong et al., 2011; Lecca et al., 2011). The MH projects to the interpeduncular nucleus (IPN) which, in turn, projects to the median and dorsal raphe nuclei in addition to other brain regions (Figure 2A) (Morley, 1986). Recent data indicate that expression of nAChRs containing the α5 and/or β4 subunits within the MH control nicotine intake such that genetic deletion of α5 nAChRs increases acute intake; whereas overexpression of the β4 nAChR subunit reduces intake and increases sensitivity to nicotine’s aversive properties (Fowler et al., 2011; Frahm et al., 2011). Thus, while the mesocorticolimbic pathway confers acute nicotine reward/reinforcement, the MH-IPN pathway may signal nicotine aversion (but see Laviolette et al., 2008). In addition, the Hb-IPN is a critical circuit for the expression of physical signs of nicotine withdrawal (Salas et al., 2009). Because (1) SNPs in nAChR subunit genes CHRNA3/A5/B4 are associated with alcohol dependence phenotypes, (2) these genes are robustly expressed in the Hb-IPN circuitry, and (3) α3β4 ligands modulate ethanol consumption in rodent models, future studies should explore the role of MH-IPN nAChRs in ethanol consumption and withdrawal behaviors.

## Summary

Neuronal nAChR represent novel therapeutic targets to not only treat nicotine dependence, but also alcohol dependence. The reinforcing properties of acute ethanol, are mediated, in part, by α4* nAChRs, likely expressed in DAergic neurons of the mesocorticolimbic pathway. Ethanol potentiates the response of high affinity heteromeric nAChRs to both ACh and nicotine. Thus, if ethanol increases ACh release in the VTA, DAergic neurons will be activated via nAChRs and ethanol will further potentiate this effect (Figure 2B). Chronic nicotine may upregulate these receptors and increase the reinforcing properties of ethanol. Future studies should focus on identifying additional nAChR subunits critical for ethanol effects within and outside the mesocorticolimbic circuitry.

## Conflict of Interest Statement

The authors declare that the research was conducted in the absence of any commercial or financial relationships that could be construed as a potential conflict of interest.
